# The critical role of dysregulation of antioxidant activity and carbohydrate metabolism in celiac disease 

**Published:** 2019

**Authors:** Ensieh KhalKhal, Mostafa Rezaei-Tavirani, Mohammadreza Razzaghi, Sina Rezaei-Tavirani, Hakimeh Zali, Mohammad Rostamii-Nejad

**Affiliations:** 1 *Proteomics Research Center, Faculty of Paramedical Sciences, Shahid Beheshti University of Medical Sciences, Tehran, Iran*; 2 *Laser Application in Medical Sciences Research Center, Shahid Beheshti University of Medical Sciences, Tehran, Iran*; 3 *Proteomics Research Center, Shahid Beheshti University of Medical Sciences, Tehran, Iran*; 4 *Proteomics Research Center, School of Advanced Technologies in Medicine, Shahid Beheshti University of Medical Sciences, Tehran, Iran*; 5 *Gastroenterology and Liver Diseases Research Center, Research Institute for Gastroenterology and Liver Diseases, Shahid Beheshti University of Medical Sciences, Tehran, Iran *

**Keywords:** Celiac disease, Anti-oxidant, Metabolism, Inflammation, Cell growth

## Abstract

**Aim:**

Identification of the important processes and the related genes that are dis-regulated in the celiac disease (CD) was the aim of this study.

**Background:**

Celiac disease is an autoimmune disorder which is characterized by immune reaction response mostly to wheat gluten. The gluten-free diet is the best-known treatment of the patients.

**Methods:**

Significant differentially expressed proteins (DEPs) related to the CD are extracted from a published proteomics study and are included in protein-protein interaction PPI) network analysis by Cytoscape software and its applications. The central proteins and related processes are identified and discussed.

**Results:**

Among 53 queried genes, 51 individuals were recognized by the database, and after network construction, 48 ones included in the network, and three genes remained as isolated nodes. Following 50 neighbors, the network was analyzed, and eight central genes were identified as dis-regulated elements. Related processes and the role of the central genes in celiac are discussed in detail.

**Conclusion:**

CAT, ENO1, PCK2, ACO2, ALDOOB, GALM, ADA, and ACTBADA as critical genes and Antioxidant activity, carbohydrate metabolism, inflammation, cell growth processes are highlighted as the dis-regulated individuals in CD.

## Introduction

Celiac disease, an autoimmune disease, is caused by an immune reaction response to wheat gluten or related rye and barley proteins in genetically susceptible individuals. Both genetic (human leukocyte antigen genes (HLA-DQ2 or HLA-DQ8)) and environmental factors (gluten) play a crucial role in its pathogenesis (1). The patients with this condition may present extremely polymorphic clinical manifestations, of which the most typical is chronic enteritis and malabsorption to diarrhea with weight loss (2). Osteoporosis, vitamin and mineral deficiencies, iron deficiency, and bone disease are the condition of the others that the patients may experience due to nutrition deficiency (3). Only accepted therapy for CD that most individuals respond to it is a permanent gluten-free diet (GFD) (4). Until fairly recently, the CD was regarded as rare, but recent screening studies have revealed the prevalence to be as high as 1–2% in the population (5-8).

Diagnosing CD is mainly based on the specific serological tests and the presence of a characteristic enteropathy in an intestinal biopsy (9, 10). Although specific serological tests are developed intestinal biopsy is still considered necessary for diagnosing, on the other hand, a certain level of expertise and skill for the assessment of intestinal biopsies is required, and variability in sample quality and subjective interpretation can affect on the diagnostic accuracy (11). With these diagnosis problems, earlier and reliable diagnoses for screening population is required. To achieve early diagnosis and therapy of celiac disease, detection of proteins involved in disease pathways and introducing drug targets are needed. However Several approaches such as proteomics, genomics, metabolomics, and microarray-based techniques can identify such novel celiac diagnostic biomarkers and proteins but protein-protein interaction (PPI) network analysis can be useful for detection many unknown molecular aspects of complex CD, understanding of biological processes, organization, functions of proteins, and the pathogenesis of CD, so some new diagnostic biomarkers can be introduced by study protein interaction networks (12-14). 

In this study, selected proteins merely from proteomics studies are analyzed based on the GO and PPI examination to introduce some related molecular biomarkers as a panel for celiac disease. 

## Methods

Differentially expressed genes (DEGs) related to the CD were obtained from the proteomics documents published by Simula MP et al. (15). As it is shown in tables 1 and 2, 10 up-regulated and 43 down-regulated genes were selected to include in-network creation. The network was constructed via physical interaction by STRING database application of Cytoscape software 3.7.1 (16). Since default confidence score of string was .04, the median score; 0.5, was considered. Fifty related first neighbors from STRING database were added to query genes to elevate interactions between the nodes. The main connected component was analyzed by Network analyzer to determine centrality parameters.

**Table 1:** List of 10 up-regulated proteins related to CD

R	Protein name	Gene name
1	kininogen-1	KNG1
2	serum amyloid P-component	SAP
3	ATP synthase β chain	ATP5F1B
4	enolase α acidic	Eno1
5	Proteasome subunit α type-6	PSMA6
6	actin	ACT
7	galectin-10	CLC
8	Ig mu chain C region	IGHM
9	Elongation factor 2	EEF2
10	Tryptophanyl-tRNA synthetase	WARS

**Table 2:** List of 43 down-regulated proteins related to CD

R	Pr name ( down )	Gene name
1	retinol-binding protein 4	RBP4
2	β2-glycoprotein 1	APOH
3	Vitronectin	VTN
4	Phosphoenolpyruvate carboxykinase	PCK1
5	3-hydroxy-3-methylglutaryl-CoA synthase 2	HMGCS2
6	Medium-chain specific acyl-CoA dehydrogenase	ACADM
7	Fatty Acid-Binding Protein 1	FABP1
8	Fatty acid-binding protein 2	FABP2
9	Apolipoprotein C-III	APOC3
10	Phosphoenolpyruvate carboxykinase 2	PCK2
11	Carbonyl reductase (NADPH) 1	**CBR1**
12	retinol-binding protein 2	RBP2
13	carbamoyl-phosphate synthase	**CPS1**
14	Ornithine aminotransferase	**OAT**
15	Aminoacylase-1	**ACY1**
16	Ornithine carbamoyltransferase	**OTC**
17	DnaJ homolog subfamily B member 11	DNAJB11
18	Fructose bisphosphate aldolase B	ALDOB
19	Aldose 1-epimerase	**GALM**
20	Fructose-1.6-bisphosphatase	FBP1
21	Aflatoxin B1 aldehyde reductase member 3	AKR7A3
22	Aldo-keto reductase family 1 member B10	AKR1B10
23	Glycerol-3-phosphate dehydrogenase	GPD2
24	Hydroxyacyl-coenzyme A dehydrogenase	HADH
25	Dihydroxyacetone kinase	DAK
26	Aconitate hydratase	ACO2
27	Cytochrome b5	CYB5A
28	Catalase	**CAT**
29	Sulfotransferase 1A3/1A4	SULT1A4
30	Glutathione S-transferase A1	**GSTA1**
31	GTP-binding nuclear protein Ran	**RAN**
32	Phosphatidylethanolamine-binding protein 1	**PEBP1**
33	Hypothetical protein MGC29506	**MZBI**
34	Peroxiredoxin-4	PRDX4
35	Villin 1	VIL1
36	Lamin-A/C	**LMNA**
37	Actin beta	**ACTB**
38	Actin-related protein 2/3 complex subunit 2	ARPC2
39	Guanine deaminase	GDA
40	Adenosine deaminase	ADA
41	Purine nucleoside phosphorylase	PNP
42	Calcium-activated chloride channel regulator 1	CLCA1
43	Voltage-dependent anion-selective channel protein 1	VDAC1

**Figure 1:**
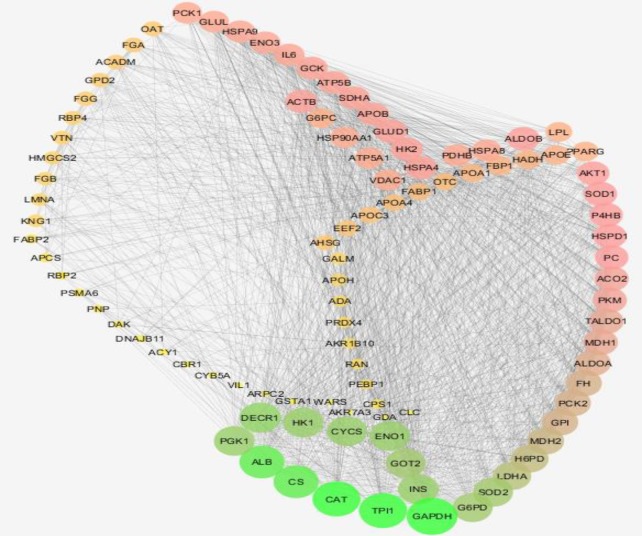
Number of 98 genes of main component and their connections is illustrated. The nodes are laid out based on degree value

10 % of top queried based on degree value, and also betweenness centrality was selected as hubs and bottlenecks respectively. The conventional hubs and bottlenecks were introduced as hub-bottlenecks nodes. Description of hubs and bottlenecks were searched via STRING databases and edited to show brief descriptions. To obtain clear relationships between the hubs and bottlenecks, their connections with their neighbors were separately investigated in the STRING database. The interaction between the genes was illustrated. 

## Results

**Figure 2:**
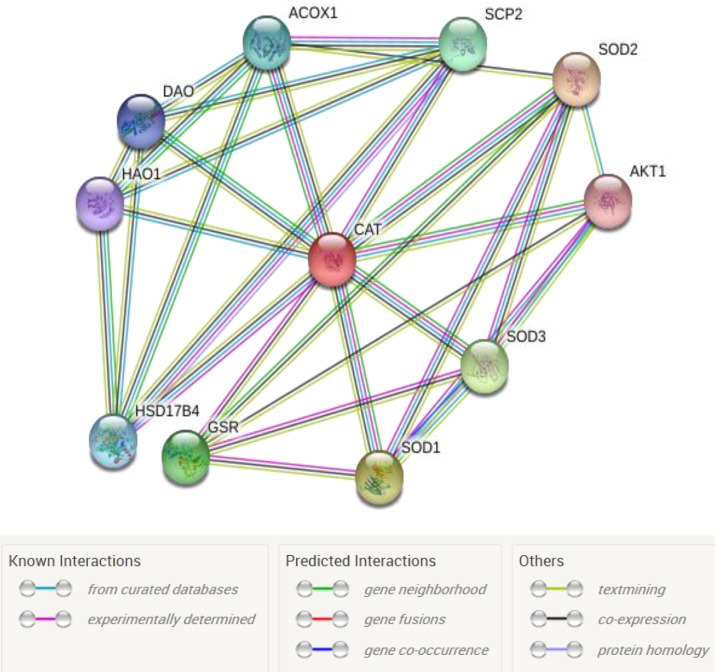
A subnetwork of CAT and its 10 neighbors is shown. Types of edges are presented in the bottom of figure

Fifty-three significant DEGs related to celiac patients were considered to construct a PPI network. Except for two genes, the other ones were recognized STRING plugin of Cytoscape software. Weak interactions characterized the network. Therefore, fifty neighbors were added to the queried genes. Since three genes were isolated, the main connected component, including 98 nodes, was created (see figure 1). About 10% of queried nodes based on the top value of degree were selected as hubs. The hub nodes and their descriptions are tabulated in table 3. As it is shown in table 4 the bottlenecks similar to the hubs are identified based on BC. Since catalase is the top hubs of query genes and also plays a role as hub-bottleneck in the network, its subnetwork including CAT and its ten neighbors is illustrated in figure 2.

**Table 3:** Five hubs of the queried genes are presented. * CAT and ALDOB are hub-bottleneck nodes. Descriptions are derived from STRING database. BC and CC refer to betweenness centrality and closeness centrality respectively

name	description	Degree	BC	CC
CAT	It is described in table4	59	1.00	0.71
ENO1	2-phospho-D-glycerate hydro-lyase; Multifunctional enzyme that, as well as its role in glycolysis, plays a part in various processes such as growth control, hypoxia tolerance and allergic responses. May also function in the intravascular and pericellular fibrinolytic system due to its ability to serve as a receptor and activator of plasminogen on the cell surface of several cell-types such as leukocytes and neurons	49	0.00	0.66
PCK2	Phosphoenolpyruvate carboxykinase [GTP], mitochondrial; Catalyzes the conversion of oxaloacetate (OAA) to phosphoenolpyruvate (PEP), the rate-limiting step in the metabolic pathway that produces glucose from lactate and other precursors derived from the citric acid cycle.	43	0.00	0.63
ACO2	Aconitate hydratase, mitochondrial; Catalyzes the isomerization of citrate to isocitrate via cis-aconitate.	40	0.00	0.60
ALDOB	It is described in table 4	38	0.50	0.61

**Table 4:** Five bottlenecks among the queried genes are shown. Descriptions are derived from STRING database

name	description	Degree	BC	CC
CAT*	Catalase; Occurs in almost all aerobically respiring organisms and serves to protect cells from the toxic effects of hydrogen peroxide. Promotes growth of cells including T-cells, B-cells, myeloid leukemia cells, melanoma cells, mastocytoma cells and normal and transformed fibroblast cells; Belongs to the catalase family.	59	1.00	0.71
GALM	Galactose mutarotase (aldose 1-epimerase); Mutarotase converts alpha-aldose to the beta-anomer. It is active on D-glucose, L-arabinose, D-xylose, D-galactose, maltose and lactose (By similarity).	16	0.50	0.51
ADA	Adenosine aminohydrolase; Catalyzes the hydrolytic deamination of adenosine and 2- deoxyadenosine. Plays an important role in purine metabolism and in adenosine homeostasis. Modulates signaling by extracellular adenosine, and so contributes indirectly to cellular signaling events. Acts as a positive regulator of T-cell coactivation, by binding DPP4. Its interaction with DPP4 regulates lymphocyte- epithelial cell adhesion. Enhances dendritic cell immunogenicity by affecting dendritic cell costimulatory molecule expression and cytokines and chemokines secretion (By similarity). Enhances CD4+ T-cell differentiation and proliferation. Acts as a positive modulator of adenosine receptors ADORA1 and ADORA2A, by enhancing their ligand affinity via conformational change. Stimulates plasminogen activation. Plays a role in male fertility. Plays a protective role in early postimplantation embryonic development (By similarity); Adenosine deaminase family	15	0.50	0.54
ACTB	Actin, cytoplasmic 1; Actins are highly conserved proteins that are involved in various types of cell motility and are ubiquitously expressed in all eukaryotic cells.	37	0.50	0.61
ALDOB*	Aldolase, fructose-bisphosphate B	38	0.50	0.61

## Discussion

A proteomic study of diseases provides useful information about the molecular mechanism of disorders which can be used in medical fields. In this regard, a large amount of data is available that can be a screen to find the most important ones (17). In a proteomic study, it is possible that above 1000 DEGs or proteins be identified (18). On the other hand, PPI interaction is a useful method to screen a large number of data to highlight the limited significant individual (19). In the present research, 53 DEGs related to CD are analyzed via this method. Results indicate that 8 of them including CAT, ENO1, PCK2, ACO2, ALDOOB, GALM, ADA, and ACTBADA play a crucial role in CD. 

It is reported that imbalance of oxidative defense system is occurred in CD due to enzymatic and non-enzymatic anti-oxidant activities. Catalase, as an anti-oxidant enzyme, plays a significant role in healthy people. Researchers confirm deficiently of catalase activity. As like catalase, it is shown SOD activity is decreased (20). In the present investigation, catalase as a down-regulated protein is introduced as a potent hub bottleneck. SOD is related to CAT in figure 2 as its neighbors.

Mitochondrial Phosphoenolpyruvate carboxykinase (PCK2) involved in the anti-inflammatory PPAR signaling pathway and downregulates in CD concerning healthy control. PPAR signaling pathway plays roles primarily in fatty acid oxidation and lipid metabolism, and secondary inflammation, and neoplastic transformation, adipogenesis, and glucose control. In the CD disease, the down-regulation of PPAR pathway could also contribute to the down-regulation of proteins involved in fatty acid and sugar metabolism observed in CD patients and direct association with the accumulation of the toxic gliadin p31-34 peptide into the cells (15, 21-24).

Aldolase B (ALDOB) is involved in metabolic pathways, Sugar metabolism, and ion transport mediators. It is down-regulated in CD patients in comparison to control group (15, 25). Deficiency of aldolase B causes hereditary fructose intolerance (HFI) and many HFI patients also affect by CD, in the other hand there is a high prevalence of CD in HFI, so there is a link between HFI and CD (26, 27).

Aldose 1-epimeras (GALM) that is known mutarotase, is a crucial enzyme of carbohydrate metabolism that catalyzes the inter-conversion of the α‐ and β‐anomers of hexose sugars such as glucose and galactose (28). Although GALM maintains the anomeric equilibrium of galactose; it plays a role in the metabolism of glucose and other sugars, and it may involve in the transport of glucose into cells (29). When GALM is inhibited, glucose reabsorption and oxidation will be blocked (30). Glucose increases in CD (31) and GALM down-regulation has occurred with respect to healthy control (15). It can be concluded that by decreasing GALM, glucose oxidation and transportation into cells will reduce. Following, the glucose concentration in the blood increases (31).

Actin beta (ACTB) is one of the two non-muscular cytoskeletal actions which it is involves in cell motility, structure, integrity, and intercellular signaling and plays critical roles in a wide range of cellular processes; including cell migration, cell division, embryonic development, and the regulation of gene expression(32-34). The common symptoms in children CD are growth failure, weight loss, short stature, and delayed puberty. Growth failure can be due to malabsorption that is in CD (35, 36). Growth deceleration results from a decline in both the rate of cell proliferation (hyperplasia) and cell enlargement (hypertrophy). In early juvenile life, body growth is primarily due to cell proliferation, leading to an increase in cell number (37, 38). Growth means getting larger, and for multicellular organisms, this is accomplished by making more cells. Cell division makes new cells to grow and also to replace old dead cells. In children, not adult, when cell division decreases, growth is reduced. It can be concluded, and cell division decreases because down-regulation of actin beta in CD patients in comparison to control group (15) and the following growth is a failure. 

Adenosine deaminase (ADA) is involved in Purine metabolism (39) and the development and maintenance of the immune system. ADA downregulation is occurred in CD patients in comparison to control group (15) following adenosine increases in CD so high levels of adenosine can exacerbate inflammation responses rather than suppressing them (40). Low levels of ADA have also been associated with pulmonary inflammation, thymic cell death, and defective T-cell receptor signaling (41, 42).

Mitochondrial aconitate hydratase (AC2) is involved in Energy production and catalyzes citrate to isocitrate in the tricarboxylic acid cycle. AC2 is downregulated in CD (15). When AC2 decreases, citrate accumulates. High concentrations of cytosolic citrate can inhibit glycolysis and ATP production (43) then glucose oxidation and ATP producing will reduce. Following, the glucose concentration in the blood increases (31). 

Enolase (ENO1), also known as phosphopyruvate hydratase, is involved in the glycolytic pathway and inflammatory process (44, 45). ENO1 level is higher in CD patients in comparison to control group (46). Besides, ENO was previously associated with human diseases (46-49). Its inhibitors have been investigated as potential treatments for cancer and infectious diseases (50).

 During the inflammatory process, enolase might be a substrate of caspase-1. Glycolysis is essential for macrophage survival and activation, reduction of glycolysis results from the cleavage of ENO1 and the glycolysis substrates, it seems to be an essential step toward cell death (44, 45)

 In pediatric patients, such as celiac disease the autoantibodies which are directed against enolase, are present in different inflammatory conditions. The B cells activation against enolase could be a systemic event (51). Antioxidant activity, carbohydrate metabolism, inflammation, cell growth, and the related genes are the important processes which are dis-regulated in CD. It seems that focus on expression change of CAT, ENO1, PCK2, ACO2, ALDOOB, GALM, ADA, ACTBADA may be a key point in CD researches.
